# Primary central nervous system lymphoma high incidence and poor survival in Finnish population-based analysis

**DOI:** 10.1186/s12885-022-09315-8

**Published:** 2022-03-03

**Authors:** Inka Puhakka, Hanne Kuitunen, Pekka Jäkälä, Eila Sonkajärvi, Taina Turpeenniemi-Hujanen, Aino Rönkä, Tuomas Selander, Miika Korhonen, Outi Kuittinen

**Affiliations:** 1 Department of Neurology, Kuopio University Hospital, PL 100, 70029 Kuopio, KYS Finland; 2 Department of Oncology, University of Oulu, Oulu University Hospital, Kajaanintie 50, 90220 Oulu, Finland; 3 School of Medicine, Institute of Clinical Medicine, Neurology, University of Eastern Finland Faculty of Medicine, Kuopio, Finland; 4 Department of Anesthesiology, University of Oulu, Oulu University Hospital, Kajaanintie 50, 90220 Oulu, Finland; 5 Department of Oncology, Kuopio University Hospital, PL 100, 70029 Kuopio, KYS Finland; 6 Kuopio University Hospital, Science Service Center, PL 100, 70029 Kuopio, KYS Finland; 7 School of Medicine, Institute of Clinical Medicine, Oncology, University of Eastern Finland Faculty of Medicine, Kuopio, Finland; 8 Department of Oncology and Radiotherapy, Kuopio University Hospital, PL 100, 70029 Kuopio, KYS Finland

**Keywords:** Pcnsl, Primary central nervous system lymphoma, Incidence, Prognosis, Survival, Cancer registry

## Abstract

**Background:**

We report here the first population-based incidence rates and prognosis of primary central nervous system lymphoma (PCNSL) in Finland.

**Methods:**

Finnish Cancer Registry data by histological diagnosis and tumor location (2007–2017) for cases with diffuse large B-cell lymphoma.

**Results:**

During 2007–2017, 392 new cases of PCNSL were reported (195 males, 197 females). The average age-adjusted incidence was 0.68/100,000 person-years. Incidence for males was 0.74/100,000 and for females 0.63/100,000, respectively. The incidence was highest, 2.93/100,000, among people aged 75–79 years. Concerning all cases in 2007–2017 the 2-year age-adjusted relative survival rate was 33% and the corresponding 5-year survival rate was 26%. Among patients under the age of 70, the age-adjusted 5-year relative survival rate increased from 36% in 2007–2012 to 43% for 2013–2017. Among patients aged 70+ the corresponding survival rates were poor, 7 and 9%.

**Conclusions:**

PCNSL incidence in Finland is among the highest reported in the world. The annual increase in incidence was 2.4%. The prognosis is still dismal, especially in elderly patients.

## Background

Primary central nervous system lymphoma (PCNSL) is a rare disease. However, several reports have described a growing incidence rate in Western countries [1–6]. The reasons for this increase are mostly unknown. A growing number of patients with immunosuppression may explain some proportion of this, but an increase is evident among immunocompetent people as well. Some environmental or lifestyle factors may explain these changes. It also seems that ethnic and genetic factors play a role, given that in the United States, the risk is different for the African American and Caucasian populations [1, 7]. The literature examining Northern Europe describes incidence rates in Sweden of 0.26/100,000 in the period 2000–2013 [8], in Norway of 0.18/100,000 during 1999–2003 [2], and in Denmark of 0.16/100,000 for 1983–1994 [3].

The prognosis for PCNSL has been poor. Current standard of care includes high-dose methotrexate containing combination chemotherapy and consolidation therapy with autologous stem-cell transplantation or whole brain irradiation [9, 10]. Several clinical trials with relatively short follow-up times have reported considerable progress in treatment outcomes among selected clinical trial populations [11–16]. However, this is in contrast to most of the population-based reports, which have demonstrated only minor improvement in long-term survival [1–6].

Here, we report Finnish Cancer Registry (FCR) [17] data concerning PCNSL incidence, age association, and survival patterns for the years 2007–2017.

## Methods

The data were extracted from Finnish Cancer Registry by histological diagnosis and tumor location. The data were ordered from the registry, and we received the completed calculations of the incidence and survival numbers, which we then analyzed. The research group did not take part in the collection of the data.

### Finnish cancer registry

The FCR, established in 1952, is a nonprofit organization funded by the Cancer Society of Finland [18] and the Finnish Institute for Health and Welfare [19]. FCR receives cancer data from hospitals and other institutions providing treatment for cancer patients, health care professionals, pathology and cytology laboratories and Statistics Finland’s cause of death data. Health care workers have a statutory obligation to report new cancer cases to the FCR. All cancer notifications since 2008 have been stored and coded by using ICD-O-3 codes (International Classification of Diseases for Oncology, Third Edition, WHO 2000, 1st revision 2013). FCR has a high level of coverage, which is shown to be over 95% for solid tumors [20]. FCR data includes no information considering patients’ performance status, received treatment or possible underlying immunosuppression, such as HIV.

### Identification of PCNSL in FCR

PCNSL diagnosis is based on histopathological confirmation from either a brain biopsy or positive cerebrospinal fluid or vitreous fluid cytology. Systemic lymphomas are excluded by whole body computed tomography scanning, bone-marrow biopsy and testicular ultrasound in males.

Inclusion criteria for the present study were histological verification of the diagnosis from a brain biopsy, or CSF or vitreous fluid cytology and flow cytometry. Because most of the primary central nervous system lymphomas present with diffuse large B-cell lymphoma histology, we restricted the analyze to this entity. For the present study, PCNSLs were defined as lymphomas located within the central nervous system (CNS; brain, eye, leptomeninges, spinal cord).

We excluded intraocular lymphomas. They are usually located intravitreally and there is no specific topography code for intravitreal location in ICD-0-3 classification. Thus, differential diagnosis between intraocular DLBCL and extraocular orbital DLBCL is not reliable.

ICD codes for topography were C70.0-C72.9 and 9680/3 for morphology. The time period was limited to the years 2007–2017, because during earlier periods, the histological subtyping was unreliable.

### Statistical analysis

The incidence rates were calculated as the number of new cases per 100,000 person-years in each year and age-adjusted as the number of new cases per 100,000 person-years using Finnish population 2015 as a reference. Incidence rates were also reported by age groups and gender. The follow-up period was calculated from the date of the diagnosis to the date of the death or the end of the year 2017. A Poisson regression model was used to study annual incidence trends during 2007–2017 and to compare incidence rates in 2007–2012 for 2013–2017. For rate parameters the 95% confidence intervals (95% CI) were also reported. Survival analyses were calculated using Kaplan-Meier method for all the data and in different age and gender categories. Age-adjusted relative survival rates were calculated as the observed patient survival (that is, overall survival) corrected for the expected survival of an equivalent group in the general population with respect to age, sex and period. This is to eliminate the effect of general changes in population survival over time. In order to obtain most up-to-date picture, denoted period analysis was used for survival calculations [21, 22]. *P*-values < 0.05 were set to indicate statistically significant results.

## Results

### Incidence

**Fig. 1:**
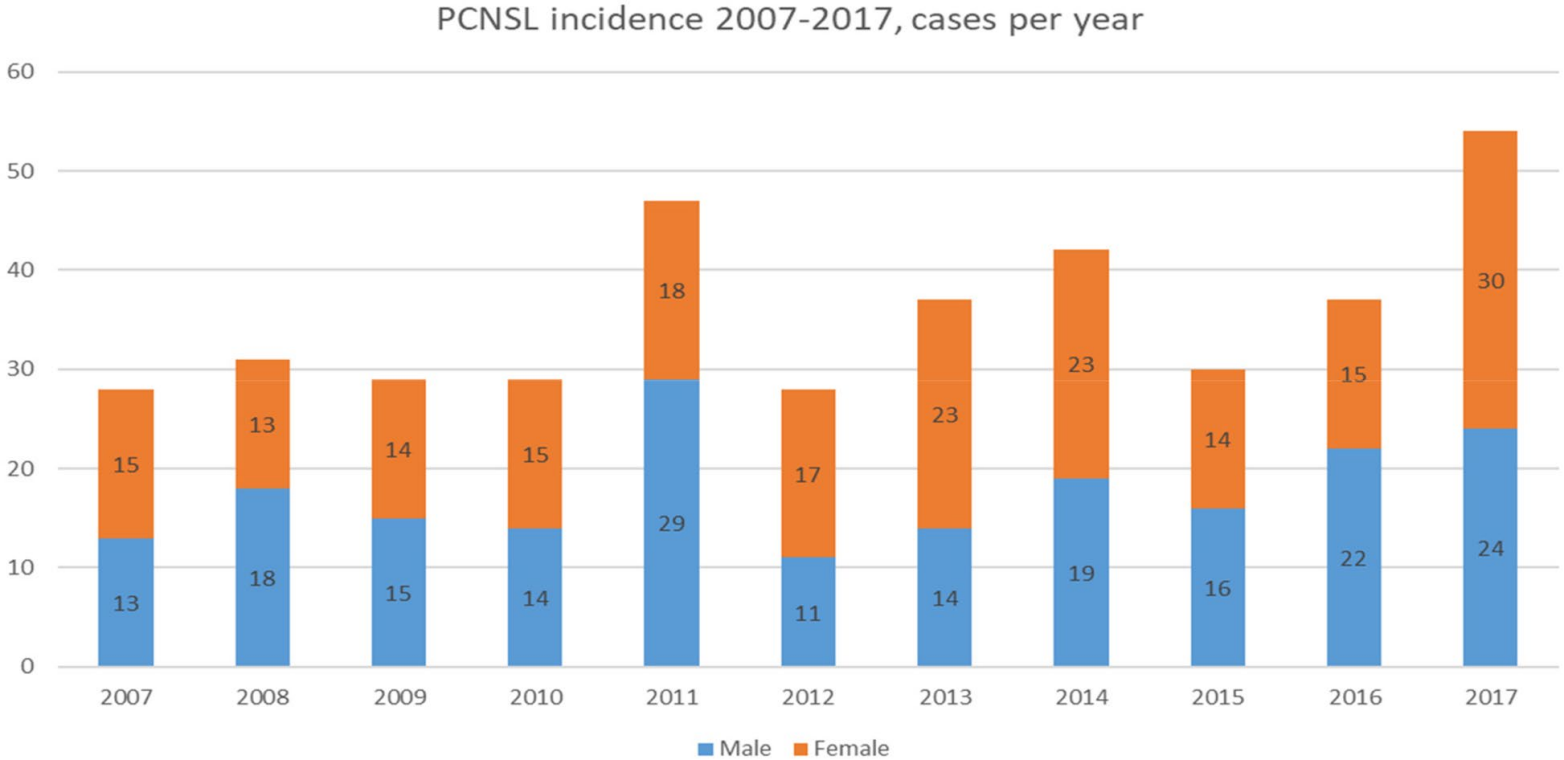
Primary central nervous system lymphoma (PCNSL) incidence in Finland 2007–2017, cases per year

**Fig. 2:**
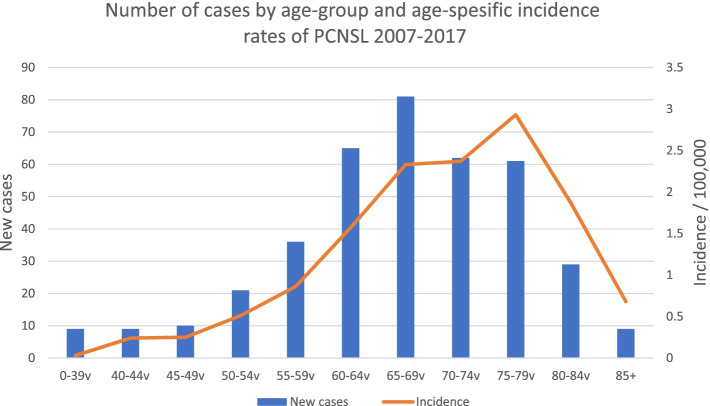
Number of cases by age-group and age-specific incidence rates of PCNSL 2007–2017

During 2007–2017, 392 (197 females, 50,3%) new PCNSL cases were reported (Fig. 1). Totally 7,1% of the patients were under 50 years old, 14,5% were 50–59, 37,2% were 60–69, 31,4% were 70–79 years, and 9,7% were aged 80+ years, as shown in Fig. 2. We discovered an increasing age-adjusted PCNSL incidence, from 0.65/100,000 person-years in 2007–2012 to 0.72/100,000 for 2013–2017. For the period 2007–2017, the annual increase in PCNSL incidence was 2.4% (95% CI: 1.9–2.9%, *p* < 0.001). The incidence was highest, at 2.93/100,000 person-years, among patients aged 75–79. The average age-adjusted incidence according to gender and time period is presented in Table 1.

**Table 1:** The average age-adjusted incidence / 100,000 person-years according to gender and time period

Period	Male, average age-adjusted incidence /100.000 (variation)	Female, average age-adjusted incidence/100.000 (variation)
2007–2009	0.70 (0.65–0.79)	0.53 (0.47–0.59)
2010–2012	0.76 (0.45–1.19)	0.61 (0.58–0.65)
2013–2015	0.65 (0.56–0.76)	0.67 (0.45–0.79)
2016–2017	0.89 (0.84–0.94)	0.75 (0.51–0.98)

Finnish population 2015 is used as a reference. In brackets is the variation of age-adjusted incidence rates between the specific individual years during the specific time period. Statistically significant difference is not observed between the time periods; *p*-values for men and women are 0.605 and 0.466, respectively.

### Survival

**Fig. 3:**
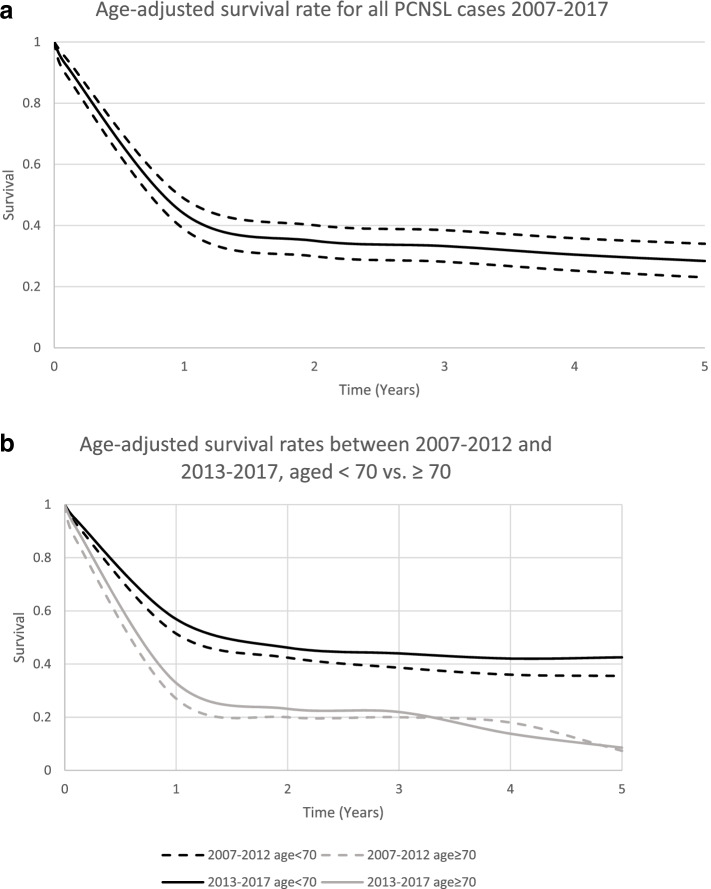
**a**) Age-adjusted survival rate for all PCNSL cases 2007–2017. **b** Age-adjusted survival rates between 2007 and 2012 and 2013–2017, aged < 70 or ≥ 70. The difference in survival between the periods was not statistically different; aged < 70 *p* = 0.621 and ≥ 70 *p* = 0.391

The 2-year age-adjusted relative survival rate in 2007–2017 was 33% (95% CI 28–38%). The corresponding 5-year survival rate was 26% (95% CI 21–32%) (Fig. 3a).

Older age was clearly related to an adverse disease outcome. In 2007–2012 the age-adjusted 2-year and 5-year relative survival rates among patients aged ≥70 years were 18% (95% CI 9–30%) and 7% (95% CI 1–27%). In 2013–2017 the corresponding rates were 23% (95% CI 14–34%) and 9% (95% CI 2–23%). *P* = 0.391.

For younger patients, under the age of 70, the 2-year age-adjusted relative survival rate increased from 42% (95% CI 33–52%) in 2007–2012 to 46% (95% CI 36–56%) for 2013–2017. The corresponding 5-year age-adjusted relative survival rates were 36% (95% CI 25–46%) and 43% (95% CI 32–52%), respectively. *P* = 0.621. Age-adjusted survival rates are displayed in Figure 3b.

## Discussion

Here, we report registry-based incidence rates and outcomes of PCNSL patients in Finland between 2007 and 2017. We discovered an increasing age-adjusted incidence, from 0.65/100,000 in 2007–2012 to 0.72/100,000 for 2013–2017. This is among the highest reported incidence rates in the world [4–6, 8]. The annual increase in PCNSL incidence was 2.4% (95% CI: 1.9–2.9%, *p* < 0.001). While the prognosis for PCNSL still seems to be poor, during the study period, a trend to minor improvement in 5-year survival rates for patients under the aged of 70, from 36% in 2007–2012 to 43% for 2013–2017, was discovered. The prognosis still seems to be poor for aged patients.

There are several registry-based reports describing an increasing incidence of PCNSL in countries with Western lifestyles [4–6, 8]. In the United States, the Surveillance, Epidemiology, and End Results (SEER) database shows that the PCNSL incidence has increased from 0.1/100,000 in the 1970s to 0.4/100,000 in 2013 [4]. In the most recent years, the increase seems to be flattening. This incidence growth has been greatest among people aged 70+, at a rate of 4.32/100,000 [23]. In Sweden, the overall incidence was 0.26/100,000 between 2000 and 2013, with an average annual increase of 4% [8]. In a population-based study from the Netherlands, the PCNSL incidence increased from 0.30/100,000 for the period 1989–1995 to 0.44/100,000 for 2009–2015. The incidence particularly increased in age groups over 60 years [6]. In Korea, the National Cancer Incidence Database shows that the average incidence of PCNSL was 0.17 between 1999 and 2009, with an annual rise of 9% [5].

The incidence in Finland seems to be among the highest reported in the world, and there seems to be no flattening of the incidence curve. However, it should be noted that in this study we had data from more recent time periods compared to studies mentioned above, which may have caused some of this difference. The etiology behind this increase is unknown. We believe that it is not explained by improved diagnostics, either. MRI scanning has already been a routine research method in Finland during the study period of 2007–2017 for patients suffering from neurological symptoms as well as for elderly patients. Moreover, in PCNSL, the symptoms progress rapidly, leading to patient deterioration, hospitalization, and diagnosis [24].

To evaluate whether the PCNSL incidence growth rate could potentially be explained by an increasing use of diagnostic imaging, we compared the incidence rates of PCNSL with the incidence rates of glioma in Finland. The incidence of glioma among the entire Finnish population increased from 6.32/100,000 in 2007–2012 to 6.82/100,000 person-years for 2013–2017, according to data received from the FCR. During the study period of 2007–2017, the median annual increase in glioma was 1.2% (95% CI: 0.3–2.3%). The PCNSL incidence growth rate was higher compared to gliomas but not statistically significant.

Immunosuppression increases the risk of PCNSL. From the FCR data, we were not able to get data about potential underlying HIV infection or other immunosuppression. However, we believe that the number of these patients is small, because HIV incidence in Finland is very low 2.5/100,000 in 2020. During 2007–2017 on average 168 HIV cases per year (146–187 cases per year) were diagnosed [25].

This fact is further supported by detailed unpublished data regarding patient comorbidities from Oulu University Hospital (2000–2018) and Kuopio University Hospital (2003–2020) districts, which shows that among all the patients diagnosed, only 6 out of 221 PCNSL cases had an immunosuppressive background; including 1 HIV and 1 organ transplantation (unpublished data from Oulu and Kuopio University Hospital registries).

Concerning the EBV incidence in Finland, in the article by Puhakka et al. [26], the seroprevalence for EBV remained unchanged in pregnant Finnish women during 1992–2012. We believe this suggests the EBV incidence in Finland has not had a major influence on PCNSL incidence either.

When analyzing the etiology behind these numbers, the difference in incidence rates between Finland and other Nordic countries is interesting. During corresponding time periods, the incidence in Finland was more than double the corresponding numbers of other Nordic countries. The genetic background of the Finnish people differs remarkably from the rest of Europe, and there are also variations in genetics between eastern and western Finns [27].

PCNSL treatment is based on intravenous high-dose methotrexate combined with other immunochemotherapeutic agents [9, 10]. In Finland a Bonn regimen [28] was mostly used as a standard of care during the study period. Simultaneously high-dose therapy and autologous stem cell transplantation were gradually adopted to routine clinical practice. In Finland radiotherapy has been used only for patients whose poor performance status prevents immunochemotherapeutic treatment or who do not respond to the treatment. Since 2008 over 80 patients have also been treated with blood-brain barrier disruption treatment (BBBD) [29, 30] in OYS, 27 patients within a phase two prospective trial and mostly outside the trial.

In our study, we found a statistically non-significant trend toward a minor improvement in survival rates of all patients. For patients under the age of 70, the 5-year age-adjusted relative survival rates increased from 36% (95% CI 25–46%) in 2007–2012 to 43% (95% CI 32–52%) in 2013–2017. In elderly patients the outcome was still poor. Among patients aged ≥70 the 5-year age-adjusted relative survival rates during 2007–2012 and 2013–2017 were 7% (95% CI 1–27%) and 9% (95% CI 2–23%), respectively.

Worldwide, the prognosis for PCNSL has been dismal, though in isolated ocular disease the prognosis is known to be better [31, 32]. Nevertheless, in recent years, several studies have reported considerable progress in treatment outcomes [11–16, 33]. For example, in the International Extranodal Lymphoma Study Group-32 (IELSG-32), the 30-month progression-free survival rate in the group treated with MATRix regimen (methotrexate, cytarabine, thiotepa, and rituximab) was 49%, compared with 23% of those treated with methotrexate-cytarabine alone and 30% of those treated with methotrexate-cytarabine plus rituximab [13]. This is in sharp contrast to the fact that in most population-based studies, the outcome is still poor, especially among older age groups [2, 5, 6, 8, 23, 34, 35]. In Sweden, there was no sign that new treatment options had translated into general survival improvements in a population-based study covering 2000–2013 [8]. In the United States, data from two national databases examining survival trends over time showed that the survival rate has increased in younger patients, while the survival rate among the elderly population has not changed in the 40 years from 1970 to 2010 [23, 34]. In the United States, the 5-year overall survival rate increased from 19,1% in 1992–1994 to 30,1% in 2004–2006 [4]. Also, in a population-based study from the Netherlands, the 5-year relative survival rate improved from 22% in 1989–1995 to 56% in 2009–2015 in patients under age 60, but at ages over 70, the corresponding rates were 3 and 6%, respectively. The overall 5-year age-adjusted survival rate increased from 11% in 1989–1995 to 30% in 2009–2015 [6]. In Korea, the overall 5-year survival rate was 29,9% between 1999 and 2007. Among patients older the age of 70, the corresponding rate was 3,7% [5]. In general, our results seem to be in line with those from the United States and Korea, where we discovered a trend toward improved survival in later periods. The reason for this improvement is probably the increasing use of combination immunochemotherapy.

In several prospective clinical studies [13, 15], the treatment results are clearly better compared to population-based studies. There may be several factors explaining this discrepancy. One is the fact that many PCNSL patients have poor performance status during diagnosis, which is usually an exclusion criterion in prospective clinical studies. Elderly patients are also rarely included. Because age and performance status are the most important prognostic factors, this means that most prospective trials recruit only the cases with the most favorable prognosis. Another possible reason for the discrepancy is that in contrast to other aggressive lymphomas, PCNSL is a disease that may relapse many years after the diagnosis [36, 37]. In a Finnish retrospective study, the long-term results for immunochemotherapy-treated PCNSL patients showed a constant pattern of relapse. Though demonstrating a favorable 2-year progression-free survival rate of 53%, after longer follow-up period, only 1 patient out of 54 remained in remission at 60 months [36]. This was discovered also in a Japanese study with a follow-up for 14 years [38]. Because prospective trials are usually reported with limited follow-up time, a considerable number of patients will relapse after follow-up. Getting reliable insight into treatment efficacy a 10-year follow-up, at least, is mandatory. In a population-based setting, new treatment options have not yet translated into major general survival improvements, although the presence of long-term survivors among fit patients is encouraging.

## Conclusions

This study provides the first population-based estimates of PCNSL incidence and survival rates in Finland. A higher PCNSL incidence in comparison to other Nordic and European countries was discovered, which is unlikely to be caused by improved diagnostics. The change in age distribution towards older age may explain part of this. We found minor improvements in PCNSL survival during the study period, probably due to improved treatment options, although the survival of aged patients is still dismal.

We discovered a high incidence rate, but if some systemic error exists, we consider it would more likely be an underestimation than an overestimation. To get reliable data, we focused only on patients with diffuse large B-cell lymphoma, which excludes the few cases with miscellaneous histologies. Only biopsy-proven cases were included, and the fact that all aged patients with a declining physical performance status will not proceed to diagnostic biopsy would further underestimate the incidence rate. Cases with eye involvement only were also excluded. There is however a chance that the difference in survival rates between the time periods is due to random variation.

## Data Availability

Finnish Cancer Registry delivered the material. The data that support the findings of this study are available from Finnish Cancer Registry but restrictions apply to the availability of these data, which were used under license for the current study, and so are not publicly available. Data are however available from the authors upon reasonable request and permission of Finnish Cancer Registry.
